# Photoperiod Management in Farm Animal Husbandry: A Review

**DOI:** 10.3390/ani15040591

**Published:** 2025-02-18

**Authors:** Chenyang Li, Hang Shu, Xianhong Gu

**Affiliations:** 1 State Key Laboratory of Animal Nutrition and Feeding, Institute of Animal Science, Chinese Academy of Agricultural Sciences, Beijing 100193, China; chenyang960521@gmail.com (C.L.);; 2 College of Animal Science and Technology, China Agricultural University, Beijing 100193, China; 3 AgroBioChem/TERRA, Precision Livestock and Nutrition Unit, Gembloux Agro-Bio Tech, University of Liège, 5030 Gembloux, Belgium

**Keywords:** photoperiod, animal welfare, feeding environment, circadian rhythm, hormone secretion

## Abstract

This review aims to examine the effects of the photoperiod on farm animals and to provide insights into how lighting management can optimize production performance, reproduction, and welfare. The production performance of farm animals is influenced by a variety of factors, such as diet, breed, and environment. Among these, lighting is a crucial component of the feeding environment. With the advancement of intensive farming, lighting measures are increasingly receiving attention. The photoperiod regulates the biological rhythms of animals and affects the secretion of hormones within the animal’s body, particularly melatonin. Melatonin regulates the secretion and release of several other hormones through various pathways, such as growth hormone, prolactin, and gonadotropins. Therefore, the environmental light cycle participates in a variety of physiological activities within animals. An appropriate photoperiod can enhance the production performance, reproduction performance, and welfare conditions of farm animals. Choosing the appropriate lighting duration based on different animals, physiological stages, and production purposes can enhance the economic benefits of farms. In this review, we summarized the recent findings on the impact of photoperiods in different farm animal feeding environments on animal husbandry, although research on the suitable photoperiod for some animals might be outdated and is also discussed in this article. For lactating dairy cows, calves, poultry, pigs (excluding boars), and rabbits, continuous light exposure exceeding 12 h per day can be implemented to enhance growth and production performance. In contrast, for boars and goats, daily light exposure should be limited to less than 10 h to optimize reproductive and productive efficiency. Overall, this review aimed to provide theoretical support for research on the optimal photoperiod for farm animals.

## Simple Summary

The study reviews how light exposure, specifically the photoperiod, influences the health, behavior, and productivity of farm animals, including dairy cows, poultry, pigs, rabbits, and goats. Photoperiod refers to the balance of light and darkness animals experience in a day, shaping their biological rhythms and affecting hormone production. Adjusting the photoperiod can improve milk yield in cows, enhance egg production in poultry, and optimize growth and reproductive performance in pigs and rabbits. However, the response to light varies across species, requiring tailored lighting strategies for each. This review highlights that appropriate photoperiod management not only enhances farm efficiency but also supports animal welfare by aligning light exposure with the animals’ natural biological needs. These findings provide valuable insights for improving livestock management practices and ensuring sustainable food production.

## 1. Introduction

The production performance of farm animals is influenced by various factors, including diet, breed, and environment. Among these, lighting is a crucial component of the rearing environment, affecting animal behavior and physiology [1,2,3]. The light environment consists of three elements: photoperiod, light intensity, and light wavelength [4,5]. Climate and geographic location, particularly latitude, significantly impact the photoperiod experience of animals. In regions closer to the equator, animals are exposed to relatively consistent light durations throughout the year, whereas those in higher latitudes experience more pronounced seasonal variations in daylight. These seasonal fluctuations in photoperiod can significantly affect animals’ biological rhythms, including their reproductive cycles and growth patterns. With the advancement of intensive farming, lighting measures are increasingly receiving attention, especially as they are influenced by the geographic and seasonal factors that vary across the globe. The photoperiod regulates the biological rhythms of animals and affects the secretion of hormones within the animal’s body, particularly melatonin (MEL). MEL regulates the secretion and release of several other hormones through various pathways, such as growth hormone (GH), prolactin (PRL), and gonadotropins. For light environments in intensive farming, changing the light intensity or wavelength may require changing the light source, whereas modifying the photoperiod is relatively easy to achieve.

**Figure 1:**
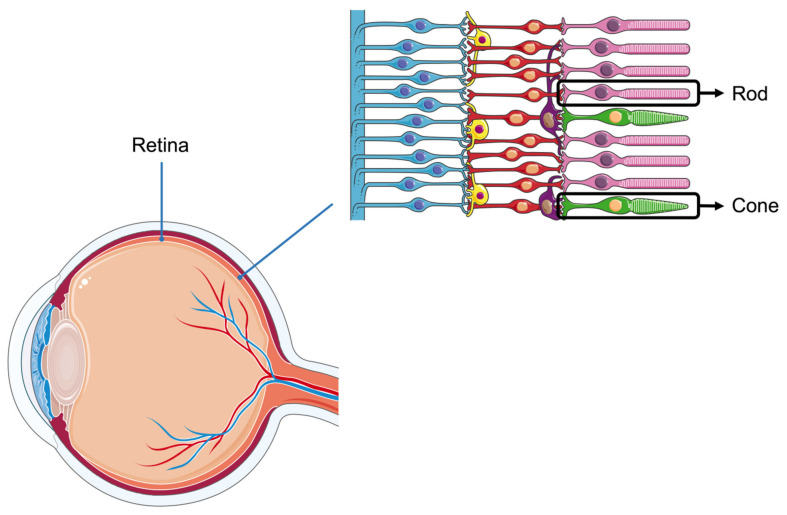
Structure of the retina. There are two types of photoreceptor cells in the retina, rod cells and cone cells. Light signals are converted into biological signals by the retina.

In the retinas of animal eyes, there are two types of photoreceptor cells: cone and rod (Figure 1). The former is sensitive to bright light, while the latter is sensitive to low light. The variation in these photoreceptor cells is considerable among different animals, hence, each species possesses its unique monochromatic or polychromatic vision. The visual systems of cattle, poultry, and pigs have distinct characteristics. Cattle have a wide field of vision and can perceive blue and yellow but have limited ability to distinguish red and green [6,7]. Poultry possess a highly developed visual system, enabling them to detect ultraviolet light and a broad spectrum of colors, including red, green, and blue [8,9]. In contrast, pigs have relatively weak vision, primarily distinguishing blue and green while exhibiting poor discrimination of red and yellow [10,11]. When the animal retina perceives light, the photoreceptor cells convert light signals into biological signals, influencing the organism’s neuroendocrine system, particularly the hypothalamic–pituitary–gonadal (HPG) axis, thereby affecting circadian rhythms and other physiological activities [12].

Light signals are converted into chemical signals, influencing the HPG axis and regulating physiological functions. Effective light management plays a crucial role in optimizing reproduction and metabolism in livestock. In broilers, extended light exposure accelerates sexual maturity, as seen in Ross broilers [13]. Similarly, in swine, prolonged lighting (23L:1D) enhances metabolic capacity and increases average daily gain (ADG) [14]. However, photoperiod effects vary by species. In short-day breeders like dairy goats, reduced light exposure triggers earlier estrus, promoting mating and pregnancy [15].

This review aims to investigate the effects of photoperiod on farm animals and how optimizing light exposure can enhance their production performance. To conduct this review, we systematically searched multiple academic databases, including Web of Science, PubMed, and Google Scholar, using relevant keywords related to photoperiod, livestock production, and physiological responses. We included peer-reviewed studies from the past three decades, with some classical studies extending beyond this timeframe, that examined the impact of light duration on growth, production performance, and reproduction. Overall, research on the effects of the photoperiod has been more extensive for dairy cows, broilers, laying hens, ducks, geese, turkeys, pigs, rabbits, goats, and horses. By synthesizing these findings, we explored the mechanisms through which the photoperiod influences animal physiology. We hypothesize that optimizing photoperiod management can improve production efficiency while maintaining animal welfare. This study provides a scientific foundation and practical guidance for enhancing lighting strategies in intensive livestock farming.

## 2. Dairy Cows

The season is a complex factor affecting milk production in cows, including temperature, humidity, light, vegetation, etc. Photoperiod is one of the important factors in light that cannot be ignored. Winter light hours are the shortest, therefore cows’ milk production will be at a low level without artificially extended light hours in winter [16]. Milk yield increases by 1 to 3 L/d when light hours are extended in winter [17]. In dairy farming, 16 to 18 h of light in a day is generally referred to as the long daily photoperiod (LDPP), while 8 h of light or less is generally referred to as the short daily photoperiod (SDPP). Studies on the effect of photoperiod on milk yield were first reported by Peters et al. [18], who reported that the milk yield of cows increased when light hours were extended to 16 to 18 h (16L:8D to 18L:6D) [19,20], but due to limited technology at that time, the mechanism was not clearly explained.

**Figure 2:**
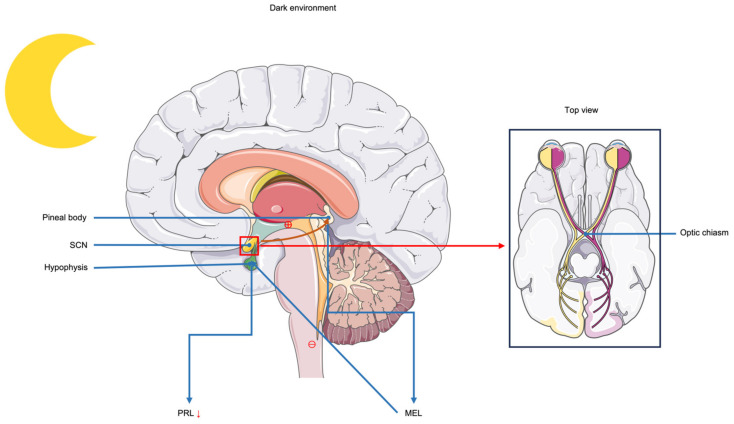
Inhibitory effect of melatonin on prolactin. When the environment is dark, nerve impulses generated in the suprachiasmatic nucleus (SCN) act on the pineal gland to promote its secretion of melatonin (MEL). MEL has an inhibitory effect on the pituitary gland and reduces prolactin (PRL) secretion. When the environment is bright, MEL secretion decreases, the inhibitory effect on the pituitary gland decreases, and PRL secretion increases. The arrows in the figure indicate a reduction in PRL secretion, with “⊖” representing inhibition of the process and “⊕” representing promotion of the process.

There are many reasons why extended light increases milk production in cows, with light affecting hormone production in cows being the main reason. Several hormones are involved in lactation in cows, such as PRL, estrogen, and GH. The role of PRL in dairy cows is to promote somatic cell proliferation and regulate mammary gland development and lactation initiation [21,22]. On the one hand, PRL levels in cows increase when they are exposed to LDPP (18L:6D) [23], and thus milk yield increases. On the other hand, when stimulated by light, light inhibits MEL secretion from the pineal gland, causing MEL levels to be low during the day and high at night [23,24], and it has been shown that MEL signaling can inhibit PRL secretion [25]. Figure 2 shows the inhibitory effect of MEL on PRL.

However, SDPP (8L:16D) increased the milk production of dry cows in their next lactation by 10% relative to dry cows in an LDPP (18L:6D) environment [23,26,27,28]. The reason can be three-fold. Firstly, the shortening of the light period during the dry period was found by some studies to increase the dry matter intake (DMI) [27,28]. Contrastingly in a study by Crawford et al. [29], there was no difference in DMI between SDPP (8L:16D)- and LDPP (18L:6D)-treated cows during the dry period. Therefore, the changes in DMI may not be the only reason for the increased milk yield during the next lactation. Secondly, cows were exposed to an SDPP (8L:16D) during the dry period, which promoted mammary gland development [30,31]. SDPP (8L:16D) also increased the expression of the PRL receptor gene [27]. Finally, the mRNA expression of insulin-like growth factor-2 was increased in cows exposed to SDPP (8L:16D) [31]. It has been reported that insulin-like growth factor-1 could improve mammary gland development and lactation by promoting cell proliferation and inhibiting apoptosis [32], hence the growth of IGF-2 mRNA expression in SDPP (8L:16D) promoted the mammary gland development in cows and contributed to increasing milk yield in the next lactation [31].

Milk composition indicators mainly include milk fat rate, milk protein rate, somatic cell score, and urea nitrogen, which are directly related to milk quality. Milk composition is influenced by a variety of factors, and the main ones currently recognized include breed, parity, lactation stage, calving period, season, temperature, management practices, etc. [33]. In 1999, Miller et al. [34] suggested that photoperiodic management does not affect milk composition. However, a lot of evidence has suggested that the composition of milk produced by cows is closely related to the duration of light exposure [35,36,37]. For instance, an early study found that LDPP increased milk fat yield in dairy cows by 0.3% [38]. Similarly, Lim et al. [39] found that the milk fat yield of cows increased with an increase in lactation under LDPP. However, there were also different findings that dairy cow milk fat yield decreases under LDPP [17,40].

In addition, milk compositions show circadian rhythmicity. For example, higher MEL levels have been reported in milk produced at night than during the day [35]. Therefore, milk produced at night can improve sleep quality [41]. When the concentration of circulating MEL in cows increases, the lactose content of milk decreases, but the fat, protein, and casein content increases, so the milk produced at night has a better milk composition [36]. However, milk production has decreased. Similarly, if we look at the annual rhythm, the long light hours in summer and the short winter months, result in higher fat and protein content in milk during the winter months [42]. This is also the reason for the difference in milk composition between summer and winter. There is also a circadian rhythm in the absorption of fat in animals [43], which is higher at night [44]. Lacto-fatty acids are also derived from rumen microbial synthesis. Ruminal microbes and their metabolites have also been reported to exhibit circadian rhythms [45]. Using lipidomics and metabolomics studies, Teng et al. [37] found that milk from cows at night contained higher levels of various fatty acids such as stearic acid, eicosapentaenoic acid, myristic acid, and cis-9-palmitoleic acid compared to milk during the day.

Calf growth is influenced by a number of factors, such as feed intake, individual differences, health status, etc. Photoperiod can also affect feed intake [46]. When light duration was extended from 10 to 18 h, the ADG of calves increased, and LDPP (18L:6D) also increased the colostrum intake in calves [47]. For the growth and development of calves, LDPP accelerated the onset of puberty in calves [47].

We have summarized the positive effects of different photoperiods on dairy cows at various physiological stages in Table 1. This section highlights the significant impact of photoperiod on dairy cow production, including milk yield, milk composition, and calf growth. Extended light exposure (16L:8D to 18L:6D) during lactation has been shown to increase milk yield by 1–3 L/day, primarily by regulating PRL secretion. However, a SDPP (8L:16D) during the dry period enhances mammary gland development and improves milk production in the subsequent lactation. While photoperiod effects on milk composition remain inconsistent, evidence suggests that nighttime milk contains higher MEL and beneficial fatty acids, improving milk quality. Additionally, photoperiod influences calf growth, with extended light exposure accelerating feed intake, ADG, and puberty onset. Despite these findings, inconsistencies exist across studies, and further research is needed to determine optimal photoperiod management strategies for different physiological stages of dairy cows.

## 3. Poultry

Poultry are highly photoreceptive animals with a well-developed visual system, making them particularly sensitive to lighting conditions. In modern intensive farming, they are predominantly raised in controlled indoor environments without access to natural light, relying entirely on artificial illumination [48]. The effects of photoperiod on poultry have been extensively studied, particularly in broilers, where lighting schedules significantly influence growth rate, feed efficiency, and welfare. However, photoperiod management also plays a crucial role in the productivity and health of laying hens, turkeys, ducks, and geese. The following sections will explore the species-specific effects of photoperiod on different types of poultry, with a particular emphasis on broilers.

### 3.1. Broilers, Laying Hens, and Turkeys

To meet the continuously increasing demand for animal protein, the number of broilers is also on the rise. The physiological activities of animals, such as metabolism, thermoregulation, and hormone secretion, are closely linked to circadian rhythms [49], and illumination affects these rhythms [50]. Appropriate lighting can enhance the dietary intake of broilers [51,52], reduce the heterophils and lymphocytes ratio (H/L) [53], aggressive behaviors [54], and fear response [55,56] in broilers.

Lighting methods in broiler farming can be classified into continuous and intermittent illumination [52]. Numerous studies suggest that intermittent lighting improves growth performance and feed efficiency [57], but findings remain inconsistent. Compared to a 2L:2D intermittent lighting schedule, continuous lighting (8L:16D from 8 to 48 days old, then 23L:1D from 49 to 56 days old) significantly reduced feed intake, body weight gain, and carcass weight [58]. A 4L:4D intermittent schedule improved serum total protein, cholesterol levels, and bone elastic modulus compared to 2L:2D [59], while a 4L:2D schedule increased body weight gain and feed intake without affecting feed conversion ratio (FCR) [60]. However, some studies found no benefits of intermittent lighting, as 3L:1D and 5L:1D schedules did not enhance live weight or slaughter rate, whereas 22L:2D continuous lighting improved weight gain and slaughter rate, though a 3L:1D schedule enhanced feed conversion efficiency [61]. Despite its potential benefits, intermittent lighting may disturb broiler rest and elevate stress, as broilers under a 4L:2D schedule showed higher corticosterone levels than those under 18L:6D or 8L:16D [60]. Elevated stress can trigger inflammatory responses, increasing cytokine release, and blood IL-6 levels [61,62]. This also suggests that intermittent light exposure has a negative impact on broiler welfare.

Earlier research suggested that extending lighting duration could improve broiler production efficiency [63], but this also disrupts their biological clock and reduces welfare. Recognizing this, international organizations such as the European Union and the Royal Society for the Prevention of Cruelty to Animals (RSPCA) have established regulations requiring at least six hours of darkness per day to support poultry welfare. Darkness is as important as light [64], as it allows broilers to rest, reduces stress, and promotes overall health [60,65]. Studies indicate that when artificial lighting is limited to 18 h or less, with at least six hours of darkness, both production performance and welfare improve [48,60,66,67]. An appropriate light cycle also influences broiler behavior. Mimicking natural lighting conditions, such as schedules like 12L:12D or 16L:8D, positively affects behavior and increases feeding time in 5- to 6-week-old broilers [48]. Shynkaruk et al. [68] found that longer dark periods (1D, 4D, 7D, 10D) further increase feeding time. However, excessive light exposure (over 20 h per day) has negative effects, including increased inflammation (Table 2). Compared to a 12L:12D cycle, a 23L:1D cycle significantly elevates IL-1β, IL-18, IL-6, and TNF-α levels in the duodenum and serum, with histological staining revealing extensive inflammatory cell infiltration in the duodenum of broilers under prolonged lighting conditions [69].

Lighting affects the circadian rhythms of animals, and these rhythms also influence gut microbiota [72,73]. Changes in gut microbiota impact digestion and nutrient absorption [74]. Studies show that under a 1L:23D lighting environment, the abundance of Bacteroides and Alistipes in broilers is significantly lower than in those under 23L:1D and 16L:8D lighting conditions [70]. When light exposure extends from 12 to 23 h, Bacteroides abundance decreases significantly [69]. Bacteroides play a key role in gut homeostasis, anti-inflammation, and intestinal pathogen defense [75]. In contrast, prolonged light exposure increases the abundance of the *Ruminococcus_torques_group*, which may damage the gut barrier and contribute to intestinal diseases [69,76,77,78]. These findings suggest that excessive light exposure may disrupt gut microbiota balance, decreasing beneficial bacteria while increasing harmful ones. Proper lighting improves broiler production and slaughter performance, potentially by maintaining gut microbiota stability in an optimal lighting environment.

The lighting environment not only affects the production performance of broilers but also impacts the egg-laying performance of laying hens. Egg production in laying hens primarily depends on the growth of the ovaries and the developmental level of the follicles, which are influenced by the environment [79].

**Figure 3:**
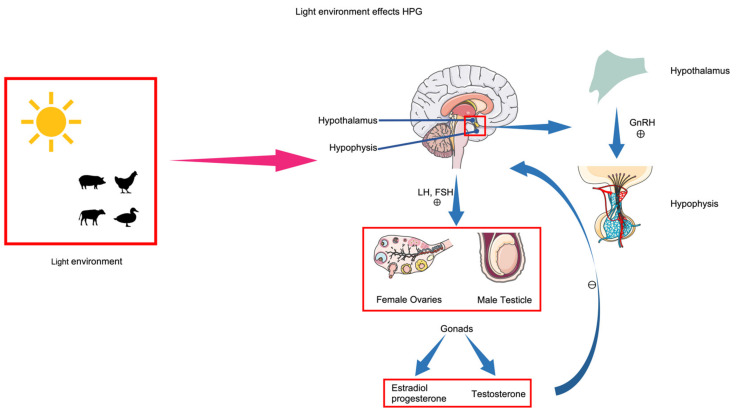
Lighting affects the hypothalamic–pituitary–gonadal (HPG) axis. The conversion of light signals into biological signals stimulates the hypothalamus to secrete gonadotropin-releasing hormone (GnRH). GnRH stimulates the pituitary gland to secrete gonadotropins (Gn). Gn acts on the gonads (testes in males, ovaries in females), which secrete sex hormones. Sex hormones, in turn, inhibit the activity of the hypothalamus and hypophysis.

Animal reproduction activities are regulated by the endocrine system, dominated by the HPG axis [80,81]. The hormonal secretion process of the HPG axis involves the hypothalamus secreting gonadotropin-releasing hormone (GnRH), which promotes the pituitary gland to secrete luteinizing hormone (LH) and follicle-stimulating hormone (FSH), acting on the gonads to ultimately promote reproduction activities (Figure 3). Increasing the photoperiod stimulates receptors in the hypothalamus, significantly increasing the secretion of GnRH, thereby promoting the secretion of LH and FSH and enhancing the reproduction performance of animals [82]. MEL, an indoleamine hormone within the body, is influenced by circadian rhythms and also regulates reproduction performance by modulating the HPG axis. In immature animals, MEL inhibits the secretion of GnRH from the hypothalamus [83], thus maintaining estrus; in mature animals, MEL has a promoting effect [84].

Research by Geng et al. [85] found that in a 12L:2D:4L:6D lighting environment, laying hens had the highest egg production rate (68%) throughout the laying period, possibly because this lighting environment mediated the optimal concentrations of PRL and LH. Compared to intermittent lighting, continuous lighting (16L:8D, 12L:12D) increased the ovarian weight, oviduct weight, oviduct length, and the number of large yellow follicles and small yellow follicles in laying hens aged 22 to 30 weeks [79], suggesting that continuous lighting is more beneficial for improving the egg-laying performance of laying hens. Different experiments have produced contradictory results, possibly due to the different ages of the laying hens. This suggests that providing different lighting environments according to the different egg-laying stages of laying hens can maximize their egg-laying performance.

Avian embryos have a certain degree of ability to receive light stimuli during embryonic development [86], and a suitable photoperiod also increases hatching performance in chickens. As early as 1972, Walter and Voitle [87] found that prolonging the light period (24L:0D) during incubation accelerated the development of chicken embryos, thereby reducing hatching time. Yameen et al. [86] also found that a 12L:12D photoperiod increased hatchability compared to 24 h of light or 24 h of darkness. In addition, 12L:12D light conditions improve the early growth performance of chicks [88]. Suitable light increases the hatching performance of poultry, which may be attributed to the fact that light leads to higher egg temperature, resulting in higher yolk temperature, which ultimately promotes early hatching [86,89].

Turkeys are also an important poultry species in livestock production. However, over the past two decades, research on the effects of photoperiod on turkeys has been limited. Studies have shown that when turkeys are reared under a 23L:1D photoperiod, their visual function significantly declines compared to those raised under a 14L:10D regimen. Turkeys exposed to prolonged light duration exhibit a higher incidence of eye diseases characterized by increased corneal curvature radius, reduced corneal refractive index, greater astigmatism, and a higher prevalence of cataracts [90]. Additionally, under the LDPP (16L:8D) conditions, the average MEL concentration in the pineal gland and retina of turkeys was significantly higher compared to turkeys raised under conventional (12L:12D) or SDPP (8L:16D) conditions [91].

### 3.2. Ducks, Geese, and Quail

The photoperiod not only influences the production and reproduction performance of broilers and laying hens but also affects the growth, physiology, and reproduction of other poultry, such as geese and ducks.

When light exposure increases, the MEL level in laying ducks at 6:00 decreases, while the mRNA expression of MEL receptors MTNR 1A and 1B increases [92]. Studies show that when light duration reaches 16 h or more, FSH and LH levels rise, promoting the development of reproductive organs and follicles [93,94]. Light exposure of 12L, 16L, and 18L increases the weight and number of large white follicles (LWF) [93]. However, Ouyang et al. [81] found that while a 24L photoperiod improved egg-laying in mountain ducks, it did not affect GnRH levels but instead inhibited gonadotropin-inhibitory hormone (GnIH) secretion, which suppresses reproduction [95]. Thus, LDPPs may enhance poultry reproduction by stimulating GnRH and inhibiting GnIH. Similarly, LDPPs improve duck growth performance. A 20L:4D photoperiod enhances the feed conversion ratio compared to 16L:8D [96] and also reduces stress while improving immune function, whereas 16L:8D is linked to higher corticosterone levels and a greater heterophil-to-lymphocyte ratio [96].

In recent decades, fewer studies have been conducted on the effects of photoperiod on goose growth performance, while the main focus has been on reproductive performance studies. The reproduction performance of geese is also influenced by lighting conditions [97]. Domestic geese are strongly seasonal birds [98], typically laying eggs in winter and spring, and ceasing to lay in summer and autumn [99]. Artificial alteration of the photoperiod can facilitate year-round production [100]. In goose farming, providing artificial supplemental lighting to extend daily light exposure leads to an earlier onset of the peak laying period and increases egg production [101]. When daily light exposure is increased from 8 h to 11 or 14 h, it promotes the reproductive activities of Hungarian White geese and upregulates the gene expression levels of OPN5, c-Fos, Dio2, and GnRH-1, maintaining reproductive activities for an extended period and resulting in high egg production performance in Hungarian White geese [102]. SDPPs (9L:15D) reduce the density of muscle fibers and the L* value (brightness) of meat color but increase the a* value (redness) of meat color in geese [103].

Photoperiod also plays an important role in quail farming, affecting their welfare, behaviors, and growth. However, fewer studies have been conducted on the effects of photoperiod on quail compared to other poultry. Longer photoperiods (16L:8D) stimulate HPA axis responses, leading to an increase in body corticosterone levels in quail, whereas SDPP (8L:16D) does not seem to elicit HPG axis responses in quail [104]. Dominchin et al. [105] also found that fecal corticosterone levels were lower in quail under SDPP (8L:16D) conditions compared to LDPP (14L:10D). However, SDPP (8L:16D) altered the behavior of Japanese quail, with a decrease in exploratory behavior but also an increase in emotional responses [106]. Similar to broilers, LDPP (18L:6D) was found to increase quail body weight in an earlier study [107]. For reproductive performance, prolonged light decreased GnIH levels in ducks, and similarly, SDPP (8L:16D) increased GnIH levels in quail follicles and ovaries, which inhibited follicular development and reduced egg production, thus affecting quail reproductive performance [108].

We have summarized the positive effects of different photoperiods on different poultry in Table 3. This section shows that photoperiod plays a crucial role in poultry production, affecting growth performance, feed efficiency, immune function, reproductive activity, and welfare. In broilers, appropriately managed lighting schedules, particularly those mimicking natural light-dark cycles, can optimize production performance while minimizing stress and inflammation. However, intermittent lighting, despite its potential benefits for feed efficiency, may negatively impact welfare by increasing corticosterone levels. In laying hens, extended photoperiods stimulate ovarian development and enhance egg production, while artificial light exposure during incubation improves hatchability and early chick growth. Similarly, prolonged lighting promotes reproductive efficiency in ducks and geese, accelerating follicular development and increasing egg yield, though excessive light exposure may disrupt hormonal balance. The limited research on quails suggests that photoperiod manipulation influences stress responses and reproductive performance, but further studies are needed. Overall, optimizing photoperiod management based on species-specific requirements and production goals is essential for maximizing poultry productivity while maintaining animal welfare. Future research should focus on refining photoperiod strategies to enhance both performance and well-being across different poultry species.

## 4. Pigs

Pigs are diurnal animals, which means they are active during the day and rest at night [109]. The European Union mandates that the lighting period in pig farming environments must be at least 8 h per day [110]. Artificial manipulation of the photoperiod, by extending the duration of light exposure, can alter pigs’ production performance and immune function, among other factors.

Weaning is a challenging process for piglets, often leading to stress that can cause intestinal diseases, reduced growth performance, and in severe cases, death [111,112]. Research has confirmed that after weaning, artificial manipulation of light to extend the illumination period to 23 h increases the feed intake and energy metabolism rate of weaned piglets [14]. Extending the lighting period from 8 to 16 h has been shown to improve the daily weight gain and immune performance of weaned piglets [113]. Similarly, Martelli et al. [110] found that compared to an 8L:16D photoperiod, a 14L:10D photoperiod increased the average daily weight gain of pigs and reduced abnormal behaviors such as standing inactive, sitting inactive, and over-exploring/over-sniffing of the floor. The reason is that extending the lighting period reduces the secretion of MEL and other neurochemicals associated with temperament, increases feeding activity, extends feeding time, and enhances digestive absorption capacity [14]. For growing and fattening pigs, extending the lighting period has a positive impact on production performance without adversely affecting carcass traits or meat quality [114].

Extending the duration of light exposure has a positive effect not only on the production performance of pigs but also on the reproduction performance of replacement gilts. Studies have found that for every additional hour of light, the age at first mating (AFM) of gilts decreases by 1.13 days [115]. Tummaruk [116] discovered that when the lighting period is extended from 11.5 h to 12.5 h, the time to first estrus in L × Y gilts is shortened by 3.04 days.

Extending the lighting period from 8 to 20 h has a promotive effect on the growth of suckling piglets and positively influences their activity without affecting the sows’ activity and nursing time [117]. However, too long lighting periods may have negative effects. Compared to a 16 h long light exposure, an 8 h light exposure during gestation is beneficial for sows to have a greater number of live piglets; however, the piglets’ birth weight is lower [113].

In boar farming, the assessment of ejaculate parameters holds significant economic importance [118]. The lighting environment has a crucial impact on the semen quality of boars, with the mechanism being the influence of light exposure duration on the body’s MEL levels. It has been found that MEL promotes testosterone production in the testes and slows down testicular aging [119]. In addition, MEL maintained normal dimethylation of spermatogonial stem cells and meiotic cells in the testes [120], which affected sperm production [121]. Knecht et al. [122] found that during short light exposure periods (8L:16D), the average semen volume of three boar breeds (PLW, P × L, D × P) was significantly higher than during long light exposure, with the most pronounced effect observed in the D × P breed. Under extreme lighting conditions (0 h of light and 24 h of light), negative impacts on boar semen concentration, semen volume, and sperm acrosome integrity were noted [123].

Lighting management in pig production, including gestating sows, gilts, boars, and growing pigs, is complex, as different lighting cycles have varying effects depending on production objectives. Studies have shown that extending light exposure (e.g., 16L:8D) for sows and piglets improves reproductive performance and growth rates, while prolonged lighting also enhances feed efficiency in growing pigs. Conversely, for boars, reducing light exposure to less than 8 h per day improves semen quality, as excessive light may negatively affect sperm production and fertility. According to the studies cited above, adopting lighting cycles tailored to the physiological stages and growth needs of pigs, such as 16L:8D for lactating sows and 8L:16D for boars, helps meet their physiological requirements, aligns with their biological rhythms, ensures overall health, and optimizes both productivity and reproductive efficiency.

We have summarized the positive effects of different photoperiods on pigs at various physiological stages in Table 4.

## 5. Rabbits

Rabbits are typically crepuscular animals and have been intensively farmed globally for wool, skin, and meat production. Observations of wild rabbits’ behavior indicate minimal activity between 10:00 and 16:00, with activity peaks occurring at 20:00 and 08:00 [124]. With the intensification of farming, most commercially produced rabbits reside in hutches with artificial lighting, making the light environment particularly important. However, research on the photoperiod for rabbit farming is relatively early-stage and primarily focused on reproduction performance. Although rabbits are nocturnal, extending the duration of light exposure may positively affect their reproduction performance. Minj et al. [125] found that, compared to short light exposure (12 h), long light exposure (16 h) environments resulted in increased levels of FSH and LH hormones in female rabbits. Quintela et al. [126] suggested that longer light exposure improved the estrus response in female rabbits, with better receptivity to artificial insemination observed after extending the light period from 8 h to 12 h six days prior to insemination. Similarly, longer light exposure (14 or 16 h) also enhances the ejaculate quality of male rabbits and improves sexual activity, increasing the sexual receptivity of female rabbits [127].

We have summarized the positive effects of different photoperiods on rabbits in Table 5.

## 6. Goats

Goats are globally renowned for their wool, with cashmere historically used to produce high-end knit fabrics [128]. Cashmere goats possess primary hair follicles (PHF), which produce coarse hair, and secondary hair follicles (SHF), which produce cashmere [134,135]. Cashmere production is influenced by genetics, nutrition, and environmental factors, with photoperiod being a key regulator of hormonal secretion. Studies show that MEL, PRL, GH, IGF-1, and thyroid horIGF-1mones play vital roles in cashmere growth [128,136,137]. MEL stimulates PRL secretion, triggering the anagen phase of hair follicles [138]. A SDPP (8L:16D) induces earlier hair follicle growth by upregulating genes associated with follicle development, with miRNAs likely playing a regulatory role [128,139]. Li et al. [66] found that under 7L:17D conditions, SHF density increased while PHF density decreased, and SDPPs elevated serum MEL levels, promoting the expression of β-catenin, BMP2, FGF5, and PDGFA, all linked to follicle development. Similarly, prolonged darkness increases MEL, PRL, and GH levels, enhances antioxidant enzyme activities (T-SOD, CAT, T-AOC), and reduces MDA concentration, thereby improving immune function and antioxidant status in goats [129,130,140].

There are fewer studies on the effect of photoperiod on the lactation performance of dairy goats. Similar to dairy cows, SDPP (8L:16D) increased lactation in dry lactation goats in the next lactation period, which may also be due to the ability of SDPP to increase PRL levels in dry lactation goats [141]. Similarly, LDPP (16L:8D) increased lactation and decreased ovulatory activity in lactating dairy goats [142].

We have summarized the positive effects of different photoperiods on goats in Table 5.

## 7. Horse

Photoperiod plays a key role in regulating various physiological aspects in horses, including coat growth, reproductive function, and hormonal regulation. Extended light exposure inhibits MEL secretion, thereby activating the molting process. Many horse owners prefer not to have thick coats on their horses, as this can impact temperature regulation in racehorses and affect the visual aesthetics of performance horses. Studies have shown that an extended photoperiod (14.5L:9.5D) helps young horses and fillies shed their winter coats [131,132]. Additionally, when the daily light duration is extended to 14.5 h for young Thoroughbreds, a significant increase in fat-free mass is observed compared to foals under natural light conditions [133]. We have summarized the positive effects of extended photoperiods on horses in Table 5.

## 8. Conclusions

Light exposure plays a critical role in regulating hormone secretion in farm animals, influencing growth, reproduction, immune function, and product quality. This review highlights species-specific responses to photoperiod manipulation. In dairy cows, prolonged light exposure enhances milk yield, while short-day photoperiods during the dry period promote mammary gland recovery. In poultry, a balanced light-dark cycle (e.g., 16L:8D) optimizes egg production and broiler growth, whereas excessive light exposure increases stress and inflammation. For pigs, extended lighting benefits sows and piglets by improving reproductive and growth performance, but reduced lighting enhances semen quality in boars. In cashmere goats, SDPPs stimulate secondary hair follicle growth and improve wool production. However, excessive or inappropriate photoperiod adjustments may disrupt circadian rhythms, induce stress, and compromise animal welfare. While current research provides valuable insights into photoperiod management, the optimal lighting duration for different species and physiological stages requires further investigation. A more precise understanding of species-specific lighting needs will enable the development of tailored photoperiod strategies to enhance productivity while maintaining animal welfare.

## Figures and Tables

**Table 1:** A summary of the positive effects of different photoperiods on cows.

Physiological Stage	Photoperiod	Effects	References
Lactation	16L:8D to 18L:6D	Milk yield ↑, PRL and MEL ↑	[21,22,23]
Dry period	8L:16D	Milk yield of next lactation ↑, mammary gland development ↑	[23,26,27,28,31]
Calves	18L:6D	ADG ↑	[46,47]

The symbol “↑” means to improve or enhance. Abbreviations: PRL: prolactin; MEL: Melatonin; ADG: average daily gain.

**Table 2:** Excessive periods of lighting have negative impacts on broilers.

Broiler Type	Days of Age ^1^	Photoperiod	Results	References
Arbor Acres	5 to 26 d	23L:1D	Intestinal injury ↑, imbalance of intestinal flora	[69]
Cobb 500	1 to 42 d	22L:2D	FBW and BWG ↑, FCR ↑, immunity and oxidative status ↓	[61]
AA	20 to 42 d	23L:1D	Altering the structure of the intestinal flora, feed-to-meat ratios ↓	[70]
Ross 308	6 to 36 d	23L:1D	H/L ratio ↑, welfare status ↓	[71]
Ross 308	8 to 35 d	24L:0D	H/T ratios ↑, AST ↑, IL-6 ↑, CORT ↑	[60]
Ross 308	15 to 45 d	20L:4D	H/L ratio ↑, stress reaction ↑	[48]

^1^ Days of age: Referring to the days of age of the broilers at the start to the end of the experiment, which reflects the entire duration of the experiment. The symbol “↑” means to enhance, and the symbol “↓” means to reduce. Abbreviations: FBW: final body weight; BWG: body weight gain; FCR: feed conversion rate; H/L ratio: heterophil to lymphocyte ratios; AST: aspartate aminotransferase; IL-6: interleukin-6; CORT: corticosterone.

**Table 3:** A summary of the positive effects of different photoperiods on poultry.

Breed	Photoperiod	Effects	References
Broilers	Intermittent illumination: 2L:2D, 4L:2D, 4L:4D, 3L:1D	Production performance ↑, FCR ↑	[58,59,60,61]
	continuous illumination: 12L:12D to 18L:6D	Production performance ↑, leg bone health ↑, stress reaction ↓, FBW and AWG ↑, welfare status ↑, hatching traits ↑, post-hatch performance ↑, feed consumption ↓	[48,60,67,88]
Laying hens	12L:12D to 16L:8D	Reproduction performance ↑	[79,85]
Laying ducks	12L:12D to 18L:6D, 24L:0D, 20L:4D	Reproduction performance ↑, FCR ↑, H/L ratio ↓, corticosterone level ↓	[81,93,94,96]
Geese	11L:13D to 14L:10D	Reproductive activity ↑	[101,102]
Turkey	16L:8D	MEL ↑	[91]

The symbol “↑” means to improve or enhance, and the symbol “↓” means to reduce. Abbreviations: FCR: feed conversion rate; AWG: average weight gain; FBW: final body weight; H/L ratio: heterophils and lymphocytes ratio; MEL: Melatonin.

**Table 4:** A summary of the positive effects of different photoperiods on pigs.

Physiological Stage or Sex	Photoperiod	Effects	References
Weaned piglets	16L:8D	ADG ↑, immunological performance ↑	[113]
Growing and fattening pigs	16L:8D	Production performance ↑	[114]
Replacement gilts	12.5L:11.5D	The time to first estrus ↓	[116]
Boar	8L:16D	Quality of semen ↑	[122]

The symbol “↑” means to improve or enhance, and the symbol “↓” means to reduce.

**Table 5:** A summary of the positive effects of different photoperiods on rabbits, goats and horses.

Animals	Breed or Physiological Stage	Photoperiod	Effects	References
Rabbits				
	Female	16L:8D	FSH and LH ↑	[125]
	Male	14L:10D to 16L:8D	Quality of semen ↑, sexual activity ↑	[127]
Goats				
	Cashmere goats	8L:16D	Wool performance ↑, immunological performance ↑, antioxidant status ↑	[128,129,130]
Horses				
	Young horses and fillies	14.5L:9.5D	Accelerated shed winter coats	[131,132]
	Young horses	14.5L:9.5D	Fat-free mass	[133]

The symbol “↑” means to improve or enhance. Abbreviations: FSH: follicle-stimulating hormone; LH: luteinizing hormone.

## Data Availability

Data sharing is not applicable.
